# Secure latent Dirichlet allocation

**DOI:** 10.3389/fdgth.2025.1610228

**Published:** 2025-07-24

**Authors:** Thijs Veugen, Vincent Dunning, Michiel Marcus, Bart Kamphorst

**Affiliations:** ^1^ Unit ICT, Strategy and Policy, TNO, The Hague, Netherlands; ^2^ Department of Semantics, Cybersecurity and Services, University of Twente, Enschede, Netherlands

**Keywords:** latent Dirichlet allocation, secure multi-party computation, Shamir secret sharing, Paillier crypto system, topic modelling

## Abstract

Topic modelling refers to a popular set of techniques used to discover hidden topics that occur in a collection of documents. These topics can, for example, be used to categorize documents or label text for further processing. One popular topic modelling technique is Latent Dirichlet Allocation (LDA). In topic modelling scenarios, the documents are often assumed to be in one, centralized dataset. However, sometimes documents are held by different parties, and contain privacy- or commercially-sensitive information that cannot be shared. We present a novel, decentralized approach to train an LDA model securely without having to share any information about the content of the documents. We preserve the privacy of the individual parties using a combination of privacy enhancing technologies. Next to the secure LDA protocol, we introduce two new cryptographic building blocks that are of independent interest; a way to efficiently convert between secret-shared- and homomorphic-encrypted data as well as a method to efficiently draw a random number from a finite set with secret weights. We show that our decentralized, privacy preserving LDA solution has a similar accuracy compared to an (insecure) centralised approach. With 1024-bit Paillier keys, a topic model with 5 topics and 3000 words can be trained in around 16 h. Furthermore, we show that the solution scales linearly in the total number of words and the number of topics.

## 1 Introduction

Topic modelling is a set of techniques that can discover abstract topics over a large set of textual documents. This is useful when there is a lot of textual data that needs to be analyzed and manual analysis is infeasible. Topic modelling can help to categorize and filter the data or to find related documents. Research until now has focused on centralized datasets, where the training data is available in one database. It is possible that certain private databases contain valuable textual data for a topic model that data holders are unwilling to share. There are two main reasons why data can be too sensitive to share: either commercially sensitive, or personal information that is privacy sensitive.

An example of the latter motivation occurs in the medical domain, where information on patients is generated by doctors in various different hospitals or other medical institutions. Combining the textual data from these different entities is valuable for two reasons: firstly, they often contain different types of information, which makes the input to the topic model more diverse and the resulting topic model richer. Secondly, topic models generally need a large amount of input, so combining inputs to train one larger topic model would result in a better topic model. The topic model can for example be used to categorize the textual data to enrich the structured patient data with new information and predict inpatient violence (1), detect virus outbreaks at an early stage (2), or get more insight into symptoms of certain diseases.

Privacy-Enhancing Technologies (PETs) provide a solution that retains the advantages of big data analytics of textual data and ensures privacy (or protects other kinds of sensitivity) of the analyzed documents. In the context of the GDPR, PETs contribute to data minimization—and therefore to proportionality—and to data control. In our work, we specifically focus on a PET called *Secure Multi-Party Computation (MPC*). In a nutshell, MPC allows to perform computations on data of multiple parties while keeping the inputs secret and only revealing the outcome.

Our work proposes an algorithm that enables topic modelling on distributed textual documents in a privacy-preserving way, using two MPC techniques called homomorphic encryption and secret sharing. This opens the door to new business cases that require topic models over textual personal data distributed over different entities, such as the ones previously mentioned.

### 1.1 Latent Dirichlet allocation

We focus on an existing algorithm called Latent Dirichlet Allocation to train a topic model for a set of documents. Intuitively, a topic model categorizes documents into different topics, where each document is assigned a combination of one or more topics. Furthermore, this gives insights into what words are often associated with these topics. Latent Dirichlet Allocation (LDA) is one of many topic modelling techniques. Among the most common topic modelling techniques, LDA is the most consistent performer over several comparison metrics, making it the most suitable algorithm for most applications (3). In particular, we consider LDA and use a technique called Gibbs sampling to train the model. Gibbs sampling is an iterative method to estimate latent distributions of a dataset based on observations from that dataset.

This means that we iterate over all the words in all the documents and observe what topic it most likely belongs to. With this topic, we then update the parameters of the topic model. This is done until the parameters converge to a stable representation of the topic model. There are also other methods to train latent parameters, but Gibbs sampling was chosen because it often yields relatively simple algorithms for approximate inference in high-dimensional models such as LDA (4, Figure 8).

### 1.2 Related work

Some research has already been done on privacy-preserving Latent Dirichlet Allocation. We can distinguish two lines of research: work that enables privacy-preserving LDA on centralized textual data, such that the final model does not leak information about the inputs (5), and work that enables LDA on distributed textual data, such that the information sent throughout the protocol does not leak information about the inputs (6–8).

Our work falls into the latter category and therefore distinguishes itself from the work in the former category by enabling LDA on decentralized data instead of centralized data. We present several new secure protocols to perform each step of the LDA algorithm in a privacy-preserving way. We now provide more explanation of the other works in the latter category. A comparison between our work and related work can be found in Table 1.

**Table 1:** Comparison with related work.

Paper	Accuracy	Speed	Security
YN10 (8)	Medium	Low	Medium: leaks probability distributions of topics
WTS20 (7)	Low	Medium	Medium: leaks statistics about all information
CD16 (6)	High	High	Low: leaks the complete document-topic matrix
Our work	High	Low	High: leaks just the total word count

The first work on privacy-preserving LDA on distributed data was published in 2010 by Yang and Nakagawa (8). Similar to us, they use homomorphic encryption. They use a custom protocol to draw the topics, which reveals the distributions to all parties. Additionally, they use a slightly altered version of the LDA algorithm, as do we. Whereas they argue the validity of their alteration with a notion of convergence based on the number of changes the algorithm makes, we use a more robust analysis using the perplexity score, showing that our alteration retains the quality and convergence rate of regular LDA.

Wang, Tong and Shi (7) propose a privacy-preserving LDA solution using federated learning and differential privacy. Their solution makes it possible to do local sampling, as the intermediate values are perturbed using differential privacy techniques. As their experiments show, this comes at a quality cost, as the perplexity score is higher for their solution than for regular LDA. Instead, we use homomorphic encryption to keep all information hidden, including intermediate values.

Colin and Dupuy (6) propose a solution to decentralized LDA with varying network topologies. They claim that their solution attains privacy of the textual documents, but no privacy arguments are given. In each iteration, two nodes, each holding a number of documents, exchange (and average) their local statistics. This is similar to sharing the matrix $n_{m}^{\left(k\right)}$, which we avoid in our solution for privacy reasons.

### 1.3 Our contributions

We present a novel solution for decentralized topic modelling in a privacy-preserving manner using latent Dirichlet allocation. This is the first solution that does not leak anything about the content of the documents while at the same time maintaining the accuracy of non-private versions of LDA. This way, we bridge the gap between accuracy and security in distributed LDA training by presenting a solution that is both highly accurate as well as secure. Furthermore, we present two generic, cryptographic building blocks of independent interest:

– Securely drawing a random number from a finite set without revealing the drawing probabilities, as described in Sections 3.4, 3.5.
– A generic solution to efficiently convert (multiple) additively homomorphic encrypted values to secret sharings, as described in Sections 3.6, 4.2.

### 1.4 Problem setting

In this work, we consider the scenario where the documents are not stored in a single database, but are distributed among multiple parties that want to train a joint topic model, but do not wish to simply share these documents with each other. Concretely, our goal is to mimic the existing LDA algorithm in a privacy-preserving manner while maintaining the *same* accuracy as the non-private version of the algorithm.

Suppose we have M documents, document m, 1≤m≤M, containing $N_{m}$ words. We consider the setting where we have multiple parties, each having one or more (sensitive) documents. Let K be the number of topics, and V the number of terms^1^ in our vocabulary. Let $\alpha =(\alpha_{1},\;\ldots ,\;\alpha_{K})$ be the Dirichlet hyperparameters for the topics in the topics-document distribution, and $\beta =(\beta_{1},\;\ldots ,\;\beta_{V})$ the Dirichlet hyperparameters for the terms in the terms-topic distribution. All these parameters are public.

During the distributed algorithm, we need to manage the secret matrix elements $n_{m}^{\left(k\right)}$, representing the number of words in document m that have topic k, and $n_{k}^{\left(t\right)}$, representing the number of words with term t that have topic k. Note that $\{n_{m}^{\left(k\right)}{\}}_{m\in \{1\ldots M\},k\in \{1\ldots K\}}$ is a matrix, which will be referred to as the *document-topic* matrix. Furthermore, $\{n_{k}^{\left(t\right)}{\}}_{k\in \{1\ldots K\},t\in \{1\ldots V\}}$ will be referred to as the *topic-term* matrix. The document-topic matrix can be split into M vectors, such that each party can manage and store only the vectors corresponding to its own documents. For the second matrix we need a different solution to avoid sharing sensitive data, see Section 3.

The purpose of the algorithm is to train the latent variable $z_{m,n}$, denoting the topic of the $n^{th}$ word of document m. In each iteration, for each document, and for each word within that document, a new topic is sampled for that word from a dynamic multinomial distribution. Given the word with index i=(m,n) and term t, this distribution is proportional to:

(1) $$\mathrm{Pr}(z_{i}=k)\propto \frac{n_{k,\neg i}^{\left(t\right)}+\beta_{t}}{\sum_{\tau =1}^{V}n_{k,\neg i}^{\left(\tau \right)}+\beta_{\tau }}\cdot \frac{n_{m,\neg i}^{\left(k\right)}+\alpha_{k}}{\sum_{\kappa =1}^{K}n_{m,\neg i}^{\left(\kappa \right)}+\alpha_{\kappa }},\;$$

where $n_{k,\neg i}^{\left(t\right)}$ indicates the count $n_{k}^{\left(t\right)}$, excluding the current word with index i, and similarly $n_{m,\neg i}^{\left(k\right)}$ (4). The first ratio can be roughly interpreted as the empirical probability that a word (not the current word) with topic k has term t. The second ratio can be roughly interpreted as the empirical weight of topic k in document m. The hyperparameters α and β are often called pseudo-counts (from prior belief) and contribute too.

## 2 Preliminaries

Our work leverages cryptographic techniques to ensure secrecy of the documents’ contents, while still enabling us to learn from them. There are different technologies that can be applied to enable privacy-preserving computations. In this work we use additively homomorphic encryption (AHE) (9, 10) and secret-sharing (11, 12). In its basic form, both techniques represent the messages they encrypt as integers, which is also what we follow in this work. The key difference is that AHE can be computed by a single party knowing the required information, while with secret sharing all operations need to be performed by all the parties holding the secrets. Parties can perform the linear operations on the shares individually, but for more complex operations such as multiplication and division, interaction is required between the parties. Nevertheless, for non-linear operations, secret sharing often yields more efficient solutions than AHE.

### 2.1 Additively homomorphic encryption

We denote the encryption of a message or plaintext m by [m]. We use the Paillier encryption scheme (9), which gives us the operations ⊕ and ⊗ such that:

[x]⊕[y]=[x+y] and c⊗[x]=[c⋅x],

for any public constant c, and secret messages x and y. That is, given encryptions [x] and [y] of x and y, we can obtain an encryption [x+y] of the sum x+y without decrypting the ciphertexts. The resulting ciphertext can be decrypted to yield the result, or be input for further encrypted operations.

### 2.2 Secret sharing

Secret Sharing has similar properties but works in a fundamentally different, key-less way. Suppose we have a secret s and wish to use this in a computation with a set of parties $P_{1},\;\ldots ,\;P_{n}$. The party holding the secret s can split this secret up into a number of shares $s_{1},\;\ldots ,\;s_{n}$ and send each $s_{i}$ to party $P_{i}$. We denote the sharing of s by $\langle s\rangle =s_{1},\;\ldots ,\;s_{n}$.

Each party $P_{i}$ can then compute operations for a public constant c and secret sharings $\langle x\rangle =x_{1},\;\ldots ,\;x_{n}\text{ and }\langle y\rangle =y_{1},\;\ldots ,\;y_{n}$ for secrets x and y such that:

⟨x⟩⊞⟨y⟩=⟨x+y⟩,\; c⋅⟨x⟩=⟨c⋅x⟩and⟨x⟩⊠⟨y⟩=⟨x⋅y⟩.

In this work, we use the Shamir secret sharing scheme (12), which is a *linear* secret sharing scheme. This means we can compute the linear additions and multiplications with a public constant without interaction between the parties. Multiplication of two secrets is additionally possible with communication between the parties.

## 3 Secure distributed LDA

In this section, we present the building blocks and algorithms required for securely performing the distributed LDA algorithm. To this end, we start in Section 3.1 with the required security assumptions. After that, in Section 3.2 we explain our solution for securely keeping track of the document-topic and topic-term matrices. Next we describe the main algorithm for securely performing Gibbs sampling in Section 3.3. Finally, in Sections 3.4–3.6 we respectively introduce separate building blocks for securely drawing a new topic from secret weights, computing encrypted integer weights and converting Paillier ciphertexts into Shamir secret sharings.

### 3.1 Security model

For both techniques, we assume the semi-honest setting, where each entity tries to learn as much information about the other entities’ data as possible, but does follow the steps of the protocol. For most use cases, this security model will suffice, as it is likely that honest participation will be agreed upon within a contractual agreement between the entities. Furthermore, since LDA already has some inherent privacy properties (5), it is unlikely that during execution a dishonest entity can retrieve a significant amount of information about other entities’ documents. However, we acknowledge this security model might not be appropriate for large-scale deployments with many potentially dishonest entities.

### 3.2 Tracking the matrices

As highlighted in Section 1.4, LDA essentially manages and updates two matrices: a document-topic matrix and a topic-term matrix. The document-topic matrix keeps track of the topic distribution of each document and consists of elements $n_{m}^{\left(k\right)}$, representing the portion of document m belonging to topic k. The topic-term matrix keeps track of the topic distribution of each term in the vocabulary and consists of elements $n_{k}^{\left(t\right)}$, representing the portion of term t belonging to topic k over all documents.

However, these matrices are precisely the sensitive information that completely leaks the content of the documents of a party when simply giving it away. Therefore, we need to find a secure way to store these matrices without (significantly) decreasing the accuracy of the algorithm.

A crucial observation is that during the LDA algorithm, the matrix elements $n_{m}^{\left(k\right)}$ of the document-topic matrix are only needed by the party actually holding document m. Therefore, it is not needed to maintain a complete, joint matrix of all the documents, but it suffices to let each party locally maintain a part of that matrix corresponding to only its own documents.

On the other hand, the topic-term matrix depends on the distribution over all the documents and should therefore be available to all the parties in an oblivious way. Maintaining this matrix comes down to adding to, and subtracting from, the elements in the matrix, which suggests the use of additively homomorphic encryption for this. To avoid individual parties from decrypting and learning the entries, we furthermore need *threshold* decryption (10). This ensures that a decryption can only be done if all the parties participate. Note that if we were to do this with secret sharing, each party would need to keep track of the entire matrix, which would introduce a lot of computational overhead.

### 3.3 Performing the algorithm

**Figure 1:**
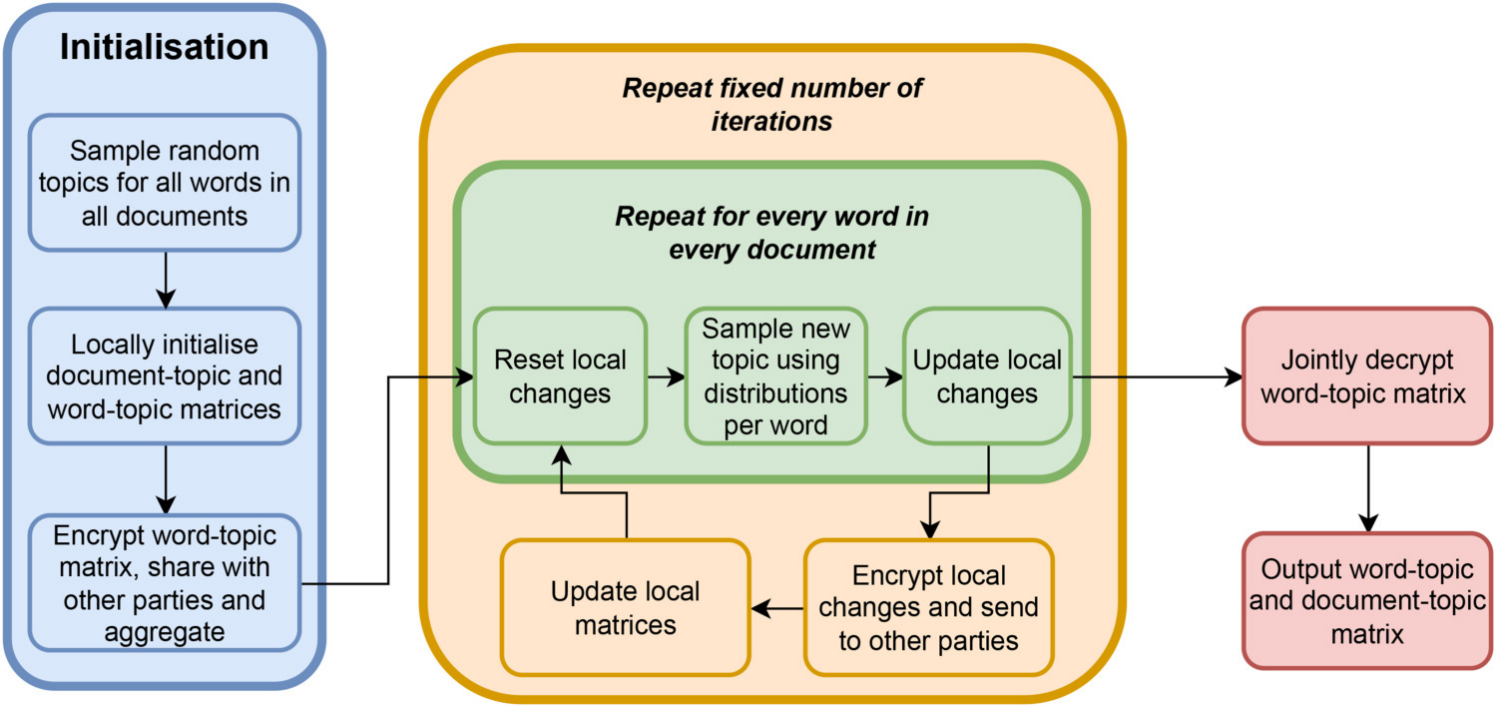
Intuitive sketch of our algorithm.

A formal description of our Secure LDA solution for securely computing the topic-term matrix $n_{k}^{\left(t\right)}$ and the document-topic matrix $n_{m}^{\left(k\right)}$ can be found in Algorithm 1. In Figure 1, we present an intuitive overview of how our algorithm works. Roughly speaking, our Secure LDA solution consists of three phases: *initialisation* (blue), *sampling* (green) and *updating* (orange). Finally, the results are decrypted in a joint decryption phase (red).

In the initialisation phase, the goal is to initialise the two matrices with a random distribution that will be refined. To this end, all the parties sample random topics for each word in each document, and use these to fill in an initial (local) view on the document-topic matrix and the topic-term matrix. Next, the parties need to build a global view of the complete topic-term matrix. To achieve this, the parties encrypt all the elements in their local topic-term matrix and combine these by sending the encrypted elements to each other and aggregate them into a global matrix by adding the (encrypted) matrices of all the parties element-wise.

After the initialisation, for a fixed number of iterations, the parties perform a *sampling* and an *updating* phase. During the sampling phase, the parties use the (secret) matrices as they are at the start of the iteration, to compute, for each word in each document, a probability distribution over the topics. The secure sampling procedure ensures that the distributions remain hidden from the parties and is outlined in Sections 3.4, 3.5. For each party, the secure sampling procedure yields a new topic for each word in each document. A party uses this information to update her local version of the encrypted topic-term matrix and local document-topic matrix.

The distribution that is drawn from is proportional to Equation 1. Note that these distributions are in an encrypted form and the actual probabilities can thus not be seen by the parties. First, we compute the encrypted weights for all the topics using the procedure presented in Section 3.5. After that, we can perform a secure draw from the encrypted weights using our novel algorithm to draw from a secret probability distribution as presented in Section 3.4. This way, the parties obtain for each word in each document a newly sampled topic. During this sampling, the parties locally keep track of the matrix updates, which means that they decrease their local counters corresponding to the matrix elements of the old word topic by one, and increase the counters for the new topic by one.

The second part of each iteration then consists of each party updating its local document-topic matrix and the parties together updating the global topic-term matrix using the locally tracked changes. To this end, each party encrypts their local changes to the topic-term matrix and sends this to all the other parties. Then the parties can simply add these encrypted counters to their encrypted topic-term matrix to get the new, consistent, topic-term matrix. The document-topic matrix can be updated locally by each party without any communication.

We observe that the LDA algorithm requires linear computations, except for the computation of the probability $\mathrm{Pr}(z_{i}=k)$ and the secure draw that uses these probabilities in the sampling step. Therefore, we perform most of the operations for tracking the topic-term matrix using AHE, and introduce a novel mechanism to switch between AHE and secret sharing in Sections 3.6, 4.2 to obtain the best performance. Concretely, we use AHE for the linear operations and only switch to (Shamir) secret sharings for securely drawing the new topics.

Typically, convergence of an LDA algorithm is checked by monitoring the changes in the model parameters, or monitoring how well the model fits a separate set of documents. In the encrypted domain, this can be quite costly to check after each iteration. Therefore, we simply iterate a sufficiently large, fixed number of times.

### 3.4 Random draw with secret probabilities

An important building block of secure LDA is a method of drawing a new topic $\tilde{k}\in \{1,\;\ldots ,\;K\}$, given secret weights $w_{k}\in N$, such that

$$\mathrm{Pr}(\tilde{k}=k)=\frac{w_{k}}{\sum_{i}w_{i}},\;\;1\leq k\leq K.$$

The new, randomly chosen topic will be revealed to party p, the holder of the current document. The intuition behind our solution is to compute *cummulative* weights $S_{k}$, k∈{1,…,K} such that $S_{k}=\sum_{i=1}^{k}w_{i}$. For notational convenience, we define an “extra” weight $S_{0}=0$. Next, the parties sample a random value r in the range $\left\{0,\;S_{K}-1\right\}$ and find between which two cumulative weights this value r lies, which then corresponds to the sampled topic. Since r is sampled uniformly at random in the total range, the probability of r precisely ending up between cumulative weights $S_{k-1}$ and $S_{k}$ is exactly $(S_{k}-S_{k-1})/S_{K}=w_{k}/\sum_{i}w_{i}$. This can be implemented with only $\log_{2}K$ secure comparisons between r and thresholds $t=S_{k}$ (with varying k) by traversing a binary tree from the root to the leaf representing the new topic. Note that our solution assumes that the weights are integers. In Section 3.5, we explain how we securely transform fractional weights into integer weights.

Formally, the parties do the following for every word w in each document:

1. The parties generate a secret random number ⟨r⟩, $r\in \{0,\;\ldots ,\;S_{K}-1\}$:
  (a) They generate a secret random number ⟨R⟩, $R\in \{0,\;\ldots 2^{\ell }-1\}$ for sufficiently large ℓ.
  (b) They securely multiply ⟨R⟩ with $\left\langle S_{K}\right\rangle$, and compute the secure truncation ⟨r⟩, where $r=\lfloor \frac{R\cdot S_{K}}{2^{\ell }}\rfloor$
2. They find $\left\langle \tilde{k}\right\rangle$, such that $S_{\tilde{k}-1}\leq r<S_{\tilde{k}}$, by repeating $\log_{2}K$ times:
  (a) Party p determines the next secret threshold ⟨t⟩ (see below).
  (b) The parties compute the secure comparison ⟨(r<t)⟩, and reveal the outcome to p.

To see that indeed a uniformly random variable r is generated, we count the number of R that lead to r=x, for $0\leq x<S_{K}$. We need $x\leq \frac{R\cdot S_{K}}{2^{\ell }}<x+1$, i.e. $\frac{2^{\ell }\cdot x}{S_{K}}\leq R<\frac{2^{\ell }\cdot x}{S_{K}}+\frac{2^{\ell }}{S_{K}}$. The number of R that satisfy this is $\left\lfloor \frac{2^{\ell }}{S_{K}}\right\rfloor$, or $\lfloor \frac{2^{\ell }}{S_{K}}\rfloor +1$. Therefore, we need $\ell \geq \log_{2}S_{K}+\kappa$, where κ is the statistical security parameter, to assure that r is statistically indistinguishable from a uniformly random variable.

The first threshold choice will be $t=K_{K÷2}$, each iteration adapting the threshold following the binary search principle. This means that if r<t, we go to the left and otherwise to the right. As the numbers $\left\langle w_{i}\right\rangle$ are secret-shared, party p needs to generate a secret-shared binary indicator vector $\left\langle \delta_{1}\rangle \ldots \langle \delta_{K}\right\rangle$, such that the threshold can be computed by $\left\langle t\rangle =\sum_{i}\langle \delta_{i}\rangle \cdot \langle w_{i}\right\rangle$. Party p is the only party that can determine the binary indicator vector, because it is the only party that is allowed to learn $\tilde{k}$.

### 3.5 Computing the integer weights

A key element of Algorithm 1 is the secure, random sampling of new topics for all of the words. As explained in Section 3.3, this is done in two steps: computing the integer weights and performing the secure draw. This subsection will introduce the steps required to compute the integer weights for Equation 1 given the matrices.

**Algorithm 1:** Protocol for performing the distributed LDA algorithm.

1. Initialisation: (a) Each party p samples a random topic for each word of all its documents. (b) Each party p sets the local counters ${\left(n_{k}^{\left(t\right)}\right)}_{p}$ and $n_{m}^{\left(k\right)}$, for each of its documents m. (c) The parties encrypt ${\left(n_{k}^{\left(t\right)}\right)}_{p}$, and securely aggregate them to $\left[n_{k}^{\left(t\right)}\right]=\left[\sum_{p}{\left(n_{k}^{\left(t\right)}\right)}_{p}\right]=\prod_{p}\left[{\left(n_{k}^{\left(t\right)}\right)}_{p}\right]$. 2. Iterate a fixed number of times: (a) For each party p do i. Party p obtains the matrix elements $\left[n_{k}^{\left(t\right)}\right]$, and sets all local counters ${\left(\Delta_{k}^{\left(t\right)}\right)}_{p}\leftarrow 0$. ii. Simultaneously choose a new topic for each word n of each document m of party p: A. Set index i=(m,n). Let $\hat{t}$ be the term of word i, and let $\hat{k}$ be the current topic of word i. Party p adjusts the local counters ${\left(\Delta_{\hat{k}}^{\left(\hat{t}\right)}\right)}_{p}\leftarrow {\left(\Delta_{\hat{k}}^{\left(\hat{t}\right)}\right)}_{p}-1$, $n_{m}^{\left(\hat{k}\right)}\leftarrow n_{m}^{\left(\hat{k}\right)}-1$. B. The parties securely sample a new topic $\tilde{k}$ for word i with matrices $\left[n_{k}^{\left(t\right)}+\Delta_{k}^{\left(t\right)}\right]$ and $n_{m}^{\left(k\right)}$ (see Section 3.5), and reveal it to party p. C. Party p adjusts the local counters: ${\left(\Delta_{\tilde{k}}^{\left(\hat{t}\right)}\right)}_{p}\leftarrow {\left(\Delta_{\tilde{k}}^{\left(\hat{t}\right)}\right)}_{p}+1$, $n_{m}^{\left(\tilde{k}\right)}\leftarrow n_{m}^{\left(\tilde{k}\right)}+1$. iii. Party p encrypts the local counters ${\left(\Delta_{k}^{\left(t\right)}\right)}_{p}$, 1≤k≤K, 1≤t≤V, and communicates them. (b) The parties update the matrix elements $\left[n_{k}^{\left(t\right)}\right]$, 1≤k≤K, 1≤t≤V, with local counts to $\left[n_{k}^{\left(t\right)}\right]\cdot \prod_{p}\left[{\left(\Delta_{k}^{\left(t\right)}\right)}_{p}\right]$. 3. The parties jointly decrypt the topic-term matrix $\left[n_{k}^{\left(t\right)}\right]$ to obtain $n_{k}^{\left(t\right)}$. 4. The parties output $n_{k}^{\left(t\right)}$ and $n_{m}^{\left(k\right)}$.

We assume we are given matrices $\left[n_{k,\neg i}^{\left(t\right)}]=[n_{k}^{\left(t\right)}+\Delta_{k}^{\left(t\right)}\right]$ and $n_{m,\neg i}^{\left(k\right)}$, the first one encrypted and the second one privately known to party p, the holder of document m. We omit the index ¬i for convenience.

To sample a new topic, first the weights have to be computed that determine the probabilities according to Equation 1, which we denote as $Pr(z_{i})\propto [w_{k}^{n}]/[w_{k}^{d}]$ for simplicity. The weights consist of numerators

$$[w_{k}^{n}]=\left[(n_{k}^{\left(\hat{t}\right)}+\beta_{\hat{t}})\cdot (n_{m}^{\left(k\right)}+\alpha_{k})\right],\;$$

and denominators

$$[w_{k}^{d}]=\left[\sum_{\tau =1}^{V}(n_{k}^{\left(\tau \right)}+\beta_{\tau })\cdot (\sum_{\kappa =1}^{K}n_{m}^{\left(\kappa \right)}+\alpha_{\kappa })\right].$$

The encrypted numerators and denominators can easily be computed by party p due to the additively homomorphic property of our encryption scheme.

The only problem is that the hyperparameters α and β are not integers, while the secret sharing scheme requires the plaintexts to be integers. For this work, we chose *symmetric* priors, meaning $\alpha_{i}=\alpha$, 1≤i≤K, and $\beta_{i}=\beta$, 1≤i≤V (see Section 5.3). We then approximate the fractions $\alpha =\frac{\alpha^{n}}{\alpha^{d}}$ and $\beta =\frac{\beta^{n}}{\beta^{d}}$, where $\alpha^{n}$, $\alpha^{d}$, $\beta^{n}$ and $\beta^{d}$ are integers. Then the numerators $w_{k}^{n}$ and denominators $w_{k}^{d}$ are converted to integers ${\tilde{w}}_{k}^{n}$ and ${\tilde{w}}_{k}^{d}$ by multiplying both with $\alpha^{d}\beta^{d}$.

Eventually, we want to obtain integer weights for the secure draw (see Section 3.4). To avoid costly secure integer divisions $\frac{{\tilde{w}}_{k}^{n}}{{\tilde{w}}_{k}^{d}}$, we multiply these fractions with $W=\prod_{k}{\tilde{w}}_{k}^{d}$ to obtain ${\tilde{w}}_{k}={\tilde{w}}_{k}^{n}\cdot \prod_{\kappa \neq k}{\tilde{w}}_{\kappa }^{d}$ as follows:

1. Party p computes the encryptions $[{\tilde{w}}_{k}^{n}]=[w_{k}^{n}\cdot \alpha^{d}\beta^{d}]=([n_{k}^{\left(\hat{t}\right)}{]}^{\beta^{d}}\cdot [\beta^{n}]{)}^{\alpha^{d}\cdot n_{m}^{\left(k\right)}+\alpha^{n}}$ and $[{\tilde{w}}_{k}^{d}]=[w_{k}^{d}\cdot \alpha^{d}\beta^{d}]=([\beta^{n}]\cdot [V]\cdot \prod_{\tau =1}^{V}[n_{k}^{\tau }{]}^{\beta^{d}}]{)}^{\alpha^{n}\cdot V+\alpha^{d}\cdot \sum_{\kappa }n_{m}^{\left(\kappa \right)}}$, which are converted to secret sharings (see Section 3.6) for efficiency reasons.
2. With one fan-in multiplication (13) the parties compute $\left\langle W\rangle =\prod_{k=1}^{K}\langle {\tilde{w}}_{k}^{d}\right\rangle$.
3. For each ${\tilde{w}}_{k}^{d}$, 1≤k≤K, they jointly compute the multiplicative inverse $\left\langle ({\tilde{w}}_{k}^{d}{)}^{-1}\right\rangle$ (14, Prot.4.11).
4. The parties compute $\left\langle {\tilde{w}}_{k}\rangle =\langle {\tilde{w}}_{k}^{n}\rangle \cdot \langle W\rangle \cdot \langle ({\tilde{w}}_{k}^{d}{)}^{-1}\right\rangle$, 1≤k≤K.

### 3.6 Converting encryptions to secret-sharings

During the execution of Algorithm 1, we need to transform the encrypted weights [w] to Shamir secret sharings ⟨w⟩ to randomly draw new topics more efficiently. Suppose we have precomputed pairs ([R],⟨r⟩), such that R contains σ more bits than w, and r=RmodN, where N, N>w, is the modulus of the Shamir secret sharing scheme. Then a conversion from [w] to ⟨w⟩ is relatively straightforward:

1. Compute [w+R]=[w]⋅[R], and (jointly) decrypt it.
2. Jointly compute ⟨w⟩=(w+R)modN−⟨r⟩.

Note that R is different from the R used earlier in Section 3.4. The pairs could be precomputed as follows:

1. Each party i generates random number $R_{i}$ that has σ more bits than w, and encrypts it.
2. Each party i computes $r_{i}=R_{i}\mathrm{mod}N$, and generates a secret sharing $\left\langle r_{i}\right\rangle$ for it.
3. Each party i sends each other party a share of $\left\langle r_{i}\right\rangle$, together with $\left[R_{i}\right]$.
4. The parties compute $\left[R]=[\sum R_{i}]=\prod_{i}[R_{i}\right]$, and $\left\langle r\rangle =\sum_{i}\langle r_{i}\right\rangle$.

We have r=RmodN, because $r=(\sum_{i}r_{i})\mathrm{mod}N=(\sum_{i}R_{i})\mathrm{mod}N$, and $R=\sum_{i}R_{i}$. It is not necessary that all parties generate a random number; it is sufficient that at least t+1 parties do.

## 4 Optimisations

During the development of the protocol, we came up with several optimisations to improve the performance. The optimisations that we implemented are described below. Additional optimisations, that were not implemented due to time constraints, can be found in the Appendix A.

### 4.1 Parallelisation of secure samplings

We combine the sampling of all new topics of one party [step 2(a)iiB], such that we can parallelise each step of the binary search (see Section 3.4), and drastically reduce the number of communication rounds. This means that the probabilities from Equation 1 are not recomputed after each single topic sampling, but only when during one iteration all words of all documents of a certain party have been assigned a new topic. This version, which we will refer to as *batched* LDA, enables us to execute all secure comparisons at the same level of the binary tree (see Section 3.4) in parallel, and significantly reduce the total number of communication rounds. The disadvantage is that the drawing probabilities are not constantly adjusted, which might lead to accuracy loss, see Section 5.4.1.

### 4.2 Multiple conversions

We have multiple conversions that can be efficiently combined into one protocol. Suppose we have weights $w_{1},\;\ldots ,\;w_{\omega }$, and corresponding pairs $\left([R_{i}],\;\langle r_{i}\rangle \right)$, 1≤i≤ω, such that $\omega \cdot (\sigma +\lceil \log_{2}w\rceil +\lceil \log_{2}n\rceil +1)<\lceil \log_{2}N\rceil$, where $\left\lceil \log_{2}w\right\rceil$ is an upper bound on the bit size of the weights, $\left\lceil \log_{2}n\right\rceil$ is the bit size of number of parties n and $\left\lceil \log_{2}N\right\rceil$ the bit size of the encryption modulus. Then the ω conversions can be combined as follows.

1. $\left[C]=[w_{\omega }+R_{\omega }]=[w_{\omega }]\cdot [R_{\omega }\right]$
2. For i=ω−1 to 1 do $\left[C]=[C{]}^{2^{\sigma +\lceil \log_{2}w\rceil +\lceil \log_{2}n\rceil +1}}\cdot [w_{i}]\cdot [R_{i}\right]$
  $\left\{C=\sum_{i=1}^{\omega }(w_{i}+R_{i})\cdot 2^{(i-1)(\sigma +\lceil \log_{2}w\rceil +\lceil \log_{2}n\rceil +1)\;}\right\}$
3. The parties jointly decrypt C and split it into $C_{1},\;\ldots ,\;C_{\omega }$, each component consisting of $\sigma +\lceil \log_{2}w\rceil +\lceil \log_{2}n\rceil +1$ bits. $\left\{C_{i}=w_{i}+R_{i}\right\}$
4. For each i, 1≤i≤ω, the parties compute $\left\langle w_{i}\rangle =C_{i}\mathrm{mod}N-\langle r_{i}\right\rangle$.

This reduces the number of decryptions by a factor ω, at the cost of some extra multiplications that combined are comparable to one decryption effort. To further reduce the number of secure additions each party could pack ω random numbers before encrypting them when precomputing ([R],⟨r⟩) pairs [see Section 3.6], which also reduces the communication effort.

## 5 Evaluation

### 5.1 Security

Because topic sampling is performed in a secure, but joint way, the parties learn the total number of words in all documents of a single party. However, nobody learns the sampling probabilities, and only the document holder learns the new topics (of the words in his documents). Our solution is secure in the semi-honest model, i.e., parties are expected to exactly follow the protocol steps, but are allowed to compute with any data that is received during execution in an attempt to gain additional insights in other parties’ data.

As we use standard building blocks, such as secure comparison and random number generation, of the MPyC platform, which is known to be secure in the semi-honest model, our computations with secret-sharings are secure too. Similarly, Paillier is known to be semantically secure, and since we use threshold decryption, encrypted information will never fall in strange hands.

Therefore, we only need to investigate the conversions from encryptions to secret-sharings, as described in Section 3.6. Because the numbers R contain σ more bits than the weights, where σ is the statistical security parameter, we know that the sum w+R is statistically indistinguishable from a large random number, and can be safely revealed. Furthermore, as each party i generates its own $R_{i}$ and $r_{i}$, the sums $\sum_{i}R_{i}$ and $\sum_{i}r_{i}$ can be considered as secret random numbers.

### 5.2 Implementation

We have implemented our secure LDA approach in Python 3.8. For the homomorphic encryption functionalities, we have used the Paillier implementation available in the TNO MPC Lab (15). This implementation is based on the distributed Paillier solution presented in (10). For the functionalities based on secret sharing, we have used the MPyC framework (16). This framework implements a number of functionalities based on Shamir secret sharing. We performed all of our experiments with three parties, but stress that our implementation also works for more parties.

### 5.3 Experimental setup

For our experiments, we used the Amazon reviews dataset presented by Ni, Li and McAuley (17). In total, this dataset consists of over 200 million reviews. However, we only used the first 150 entries. Furthermore, we split these 150 entries into three separate datasets of 50 documents for the three different parties. In total, this results in a vocabulary length of V=1492 terms and a total number of 2,965 words in the distributed corpus. For the experiments, we used 5, 10, 20, 30, 40 and 50 documents per party. As the number of words is not the same for every document, we compared the number of words over all documents for the actual experiments, which is 16, 406, 873, 1549, 2,197 and 2,965 respectively. Furthermore, we chose the symmetric priors $\alpha =\beta =\frac{1}{K}$. This corresponds to the default parameter choices in the scikit-learn implementation of LDA.

All experiments have been run on a single server running an Intel Broadwell CPU at 2.1 GHz with 4 cores and 32 GB RAM. The parties communicated via (local) HTTPS connections.

### 5.4 Performance

We evaluate the performance of our solution in terms of accuracy and runtime.

#### 5.4.1 Accuracy

In order to evaluate the accuracy of our secure LDA solution, we compare its results to the results obtained when performing a regular LDA implementation without any encryption or secret sharing. We compare both using the *perplexity* metric. This metric is standard in language modelling and is defined as $\prod_{m}p_{m}^{1/N}$. Here, $N=\sum_{m}N_{m}$ is the total number of words, and $p_{m}$ is the predictive likelihood of all words in document m (4). Perplexity is an objective metric that essentially computes the geometric mean of the log-likelihood per word in a set of observed documents. Lower perplexity scores imply a model that describes the dataset better. We have implemented and compared three versions of LDA:

– **Standard LDA:** this is a standard implementation of LDA without the use of encryption and updating the matrices after each word topic generation.
– **Batching LDA:** this version also does not use encryption, but implements a *batched* version of LDA, updating the matrices only once at the end of each pass through the entire corpus.
– **Secure LDA:** this is the solution presented in this work. It implements a privacy-preserving batched version of the LDA algorithm.

By comparing the standard- and batching versions of LDA, we can measure the impact of the adaptation we made to the algorithm. By then comparing the batching- and the secure variants, we can furthermore measure the accuracy of our privacy-preserving solution.

**Figure 2:**
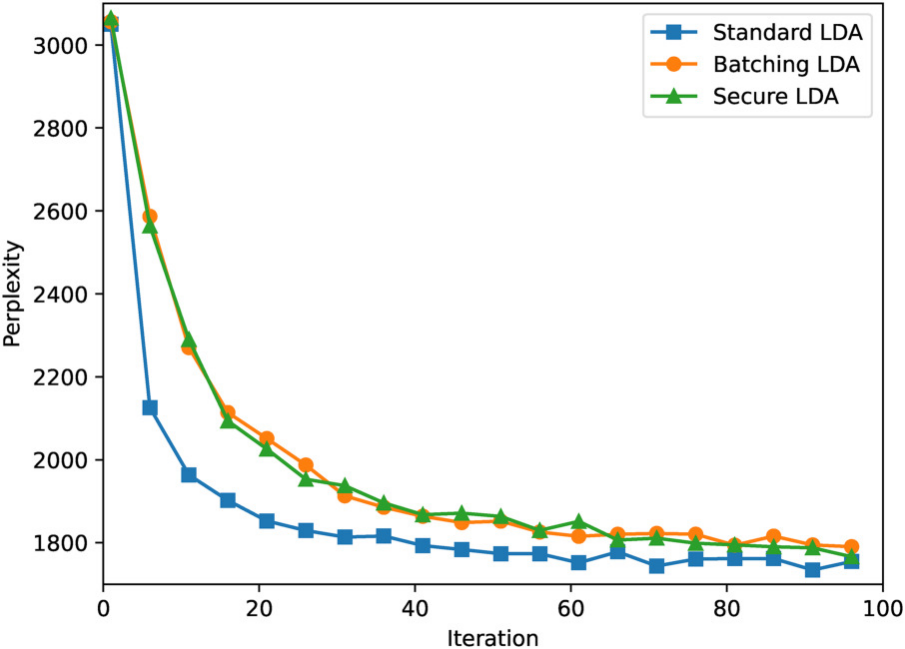
Perplexity traces of three LDA variants.

We let all three variants run for 100 iterations with two topics and 50 documents per party, which results in a total of 2965 words distributed over the parties. The results of this experiment can be found in Figure 2. We ran all versions for five times and present the average results.

As can be seen, the standard version of LDA converges faster than the batching- and secure variants. Furthermore, we see that by updating the weights after every word, the standard version generates a slightly better model. However, the differences do not seem to be significant. Finally, we observe that the secure variant shows behaviour similar to the batched plaintext variant, which strongly suggests that the use of encryption and secret sharing does not reduce the accuracy of the algorithm.

#### 5.4.2 Runtime

To see the influence of the input size and the desired complexity of the model to train, we ran benchmarks varying both the total number of words in all the documents, and the number of topics to model. We separately measured the runtime of the pre-processing step for the ciphertext conversions and performing one iteration of the secure LDA algorithm. For all benchmarks, we used a 1024-bit Paillier key^2^ for the homomorphic encryptions and a 64-bit field size for the Shamir secret shares. All parameter combinations have been tested five times and averaged.

**Figure 3:**
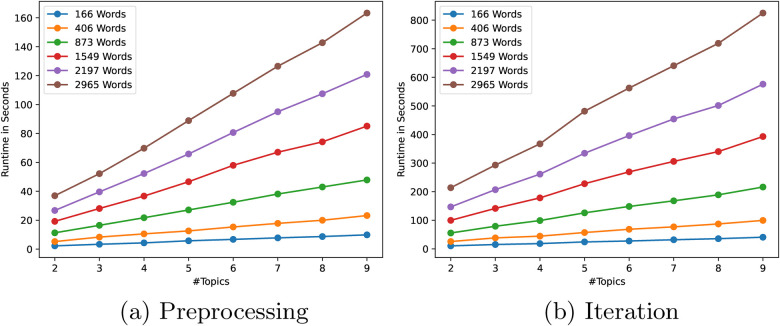
Benchmark of secure LDA in the number of topics. **(a)** Preprocessing. **(b)** Iteration.

First, we present the results for a varying number of topics for the preprocessing phase and the iteration phase in Figures 3a,b respectively. As can be seen, the amount of work for the preprocessing phase is linear in both the number N of words and the number K of topics, which is as expected as the number of tuples required per iteration is N⋅K⋅2. For the iterations, the general trend for an increasing number of topics is also linear with slightly steeper increases from 2 to 3, 4 to 5 and 8 to 9. This is explained by the fact that for the secure drawing, the number of intervals is extended by dummies to reach a power of two (either $2^{1}$, $2^{2}$, $2^{3}$, or $2^{4}$ in these experiments), which incurs an extra step in the binary search (see Appendix A.3 to avoid this). Other than that, the amount of work scales linearly in the number of topics.

**Figure 4:**
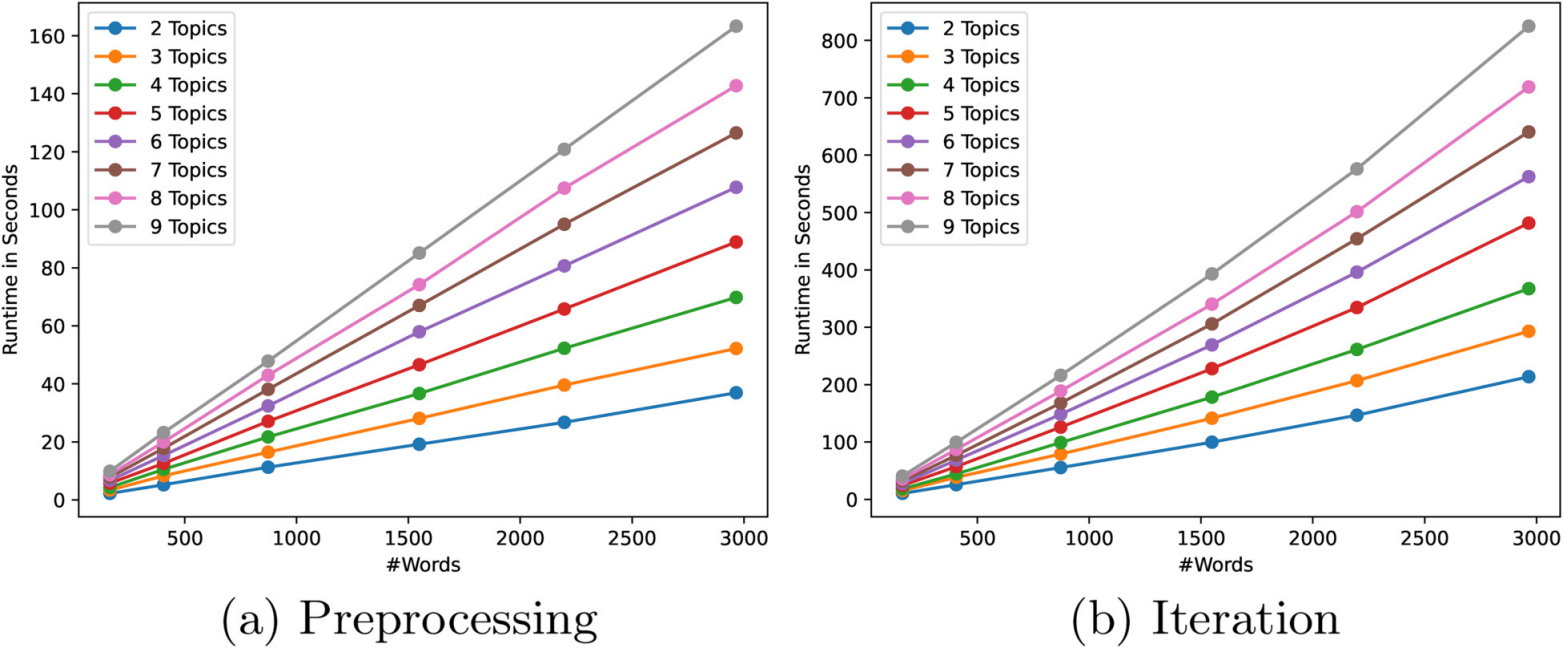
Benchmark results of secure LDA in the number of words. **(a)** Preprocessing. **(b)** Iteration.

Second, to see the influence of the input size, we also plotted the runtimes in Figure 4 against an increasing number of words over all parties. As expected, the preprocessing phase again shows a linear increase in the number of words. However, the runtime of one iteration seems to grow slightly faster than linear which might seem surprising at first as the algorithm description does not suggest exponential increase as the number of words grows. This behaviour is explained by the way we batch conversions in Section 4.2. Namely, a fixed number of weights can be converted at once, depending on the size of the Paillier modulus. As long as the number of conversions that need to be performed fits in the same number of decryptions, the runtime of an iteration grows linearly. However, if more decryptions are required in this step, the increase in runtime grows faster.

### 5.5 Comparison to prior work

As explained in Section 1.2, there are three works that also consider decentralized, privacy-preserving LDA. In Table 1, we highlight the most important differences between our works and these related works. Due to the lack of comparable runtime measurements in these works it is hard to compare our work in that regard. Instead, we turn to a conceptual comparison.

In terms of accuracy, it is unclear how the altered algorithm of (8) impacts the accuracy exactly since they do not provide metrics such as perplexity. We do know that their convergence notion influences the resulting model accuracy to some extend. Furthermore, they leak the probability distributions for the topics in every round, which is a privacy risk as this reveals information about other parties’ data. Our solution keeps $\mathrm{Pr}(z_{i}=k)$ secret throughout the entire protocol. Furthermore, they do not provide a security argument for their solution, which we do.

Due to the use of differential privacy, (7) is not able to match the accuracy of non-private LDA like we are able to do using MPC. Furthermore, this is a weaker security guarantee and might still leak some (statistical) information about the data of the other parties. This solution is, however, faster than our solution.

Finally, in (6) an approach is used where statistical information about the documents of the parties is shared in every round. This way, they are able to learn models with high accuracy and obtain a high performance at the cost of very low security guarantees as this essentially comes down to sharing your document-topic matrix.

All in all, our solution is very secure and accurate, at the cost of a lower performance. However, our solution scales linearly in both the number of words and the number of topics, which makes it scalable in practice.

## 6 Conclusions

In this work, we have presented and evaluated a fundamentally new approach to securely perform an LDA algorithm on a set of documents distributed amongst several, untrusting parties. Compared to earlier solutions, our solution provides stronger secrecy as we keep almost *everything* secret, including the topic weights. The only thing leaked in our solution is the total number of words over all documents of a party. Furthermore, we minimize the risk of leakage as the data is protected using cryptographic assumptions instead of statistical techniques like differential privacy, which might accidentally still leak some information. Furthermore, we show that the accuracy of our approach is similar to non-secure variants of the LDA algorithm.

Finally, we show that our solution scales nearly linear in the number of topics and the number of words. All in all, this makes it an attractive solution in practice, even for larger datasets.

## Data Availability

The original contributions presented in the study are included in the article/Supplementary Material, further inquiries can be directed to the corresponding authors.

## Footnotes

^1^Term refers to the element of a vocabulary, and word refers to the element of a document. A term has a particular meaning and can be instantiated by several words.

^2^From a security perspective, a 2048-bit key would have been preferable, but our primal goal was to investigate input scalability.

## Appendix A. Optimizations

We describe a few optional optimisations that were not implemented due to time constraints.

### A.1 Use of oblivious RAM

Another promising solution for securely storing and accessing the topic-term matrix is by oblivious RAM (18, 19). In the semi-honest model, a more efficient solution is to store the matrix entries somewhere, e.g. in the cloud, in an homomorphically encrypted way. Each party can query and modify entries, without the other parties noticing it.

### A.2 Avoid indicator vectors

To avoid generating indicator vectors and computing a secure inner product for each new threshold, we could decide to postpone the conversions. Given the encrypted weights, party p can first add the proper weights to determine the next threshold. Only then the encrypted threshold is converted to a secret-sharing. This does not increase the number of conversions. The transforming of fractional to integer weights might become more intensive though.

Given our parallel approach of combining all drawings of one party, we could compute all weights as follows:

1. $\left[N_{k}]=\prod_{\tau =1}^{V}[n_{k}^{\left(\tau \right)}+\beta_{\tau }\right]$
2. $\left[N]=[\prod_{k}N_{k}\right]$ {secure product}
3. $\left[\omega_{k}]=[N\cdot N_{k}^{-1}\right]$ {secure product and secure inverse}

Given $\left[\omega_{k}\right]$, where $\omega_{k}\propto \frac{1}{N_{k}}$, the weights for each term t can be computed as $[w_{k}^{\left(t\right)}]=\left[(n_{k}^{\left(t\right)}+\beta_{t})\cdot \omega_{k}\right]$ with one secure product. Using the local matrix $n_{m}^{\left(k\right)}$, these weights can be adjusted locally to document m, to cope with the factor $\frac{n_{m}^{\left(k\right)}+\alpha_{k}}{\sum_{\kappa =1}^{K}n_{m}^{\left(\kappa \right)}+\alpha_{\kappa }}$. This adjustment comes down to the exponentiation $[{\tilde{w}}_{k}]={\left[w_{k}^{\left(t\right)}\right]}^{v_{m}^{\left(k\right)}}$, where $v_{m}^{\left(k\right)}=(n_{m}^{\left(k\right)}+\alpha_{k})\cdot {\left(\sum_{\kappa =1}^{K}(n_{m}^{\left(\kappa \right)}+\alpha_{\kappa })\right)}^{-1}\cdot \prod_{\mu =1}^{M}\sum_{\kappa =1}^{K}\left(n_{\mu }^{\left(\kappa \right)}+\alpha_{\kappa }\right)$.

To generate a secret random number r, given term t and document m, the encryption $\prod_{k}[{\tilde{w}}_{k}]$ needs to be converted to a secret-sharing. During each iteration step of the binary search, the proper weights $\left[{\tilde{w}}_{k}\right]$ can be accumulated by party p to obtain the new threshold, which can then be converted to a secret-sharing for the secure comparison.

### A.3 Number of topics not a power of two

If the number K of topics is a power of two, the binary search can be easily performed. If $2^{\lambda -1}<K<2^{\lambda }$, then the number of iterations (λ or λ−1) of the binary search would disturb the uniform distribution of the randomly chosen topic. An easy way to fix this is to add $2^{\lambda }-K$ dummy values, such that the number of iterations is always λ. However, this takes more secure comparisons than strictly necessary. We describe a way to avoid these additional secure comparisons without leaking information.

1. Party p randomly chooses $2^{\lambda }-K$ different dummy indices $d_{i}\in \{1,\;\ldots ,\;2^{\lambda }\}$, $1\leq i\leq 2^{\lambda }-K$, such that $d_{1}<d_{2}<\ldots d_{2^{\lambda }-K}$. See argumentation below how this should be done.
2. k←1; u←1 {Initialise counters}
3. For i=1 to $2^{\lambda }$ do: {Compute new weights $v_{i}$}
  If $d_{u}=i$ then $v_{i}=0$; u←u+1 else $v_{i}=w_{k}$; k←k+1
4. The parties perform a binary search with weights $v_{i}$, $1\leq i\leq 2^{\lambda }$:
  – If there is only one non-dummy index remaining, party p ends the binary search.
  – In each iteration, party p constructs an indicator vector of length K (ignoring the dummy weights).

We need K to be even to avoid information leakage. E.g., for K=3 the index 2 will never be selected after one iteration, irrespective of the chosen dummy index. This means that party p has to first choose one special dummy in case K is odd that should not lead to skipping iterations (in Step 4). The question remains how the $2^{\lambda }-K$ random dummy indices (in Step 1) should be chosen, assuming K is even.

We order the K indices in K/2 pairs of consecutive numbers. We choose $(2^{\lambda }-K)/2$ random positions out of these K/2 pairs. We add two dummies to each chosen pair, just before each element of the pair. In this way, each of the K indices will have an identical probability of being chosen after λ (no dummies in the pair) or λ−1 (dummies in the pair) rounds.
